# Analysis of gene expression in the midgut of *Bombyx mori* during the larval molting stage

**DOI:** 10.1186/s12864-016-3162-8

**Published:** 2016-11-03

**Authors:** Bing Yang, Wuren Huang, Jie Zhang, Qiuyun Xu, Shoulin Zhu, Qiaoli Zhang, Brenda T. Beerntsen, Hongsheng Song, Erjun Ling

**Affiliations:** 1 Key Laboratory of Insect Developmental and Evolutionary Biology, Institute of Plant Physiology and Ecology, Shanghai Institutes for Biological Sciences, Chinese Academy of Sciences, Shanghai, 200032 China; 2 Veterinary Pathobiology, University of Missouri, Columbia, MO 65211 USA; 3 College of Life Sciences, Shanghai University, Shanghai, 200444 China

**Keywords:** Midgut, Microflora, Larva-to-larva molting, Microarray

## Abstract

**Background:**

Insects can be models for understanding human intestinal infection and pathology. Molting, a special period during which the old insect cuticle is shed and a new one is produced, is crucial for insect development. Holometabolous insects may experience several larva-to-larva moltings to become larger, a pupal molt and adult eclosion to become adults. During the larval molts, they stop feeding and become quiescent. Although the molting larvae become quiescent, it is not known if changes in microbiome, physiology, development and immunity of midguts occur.

**Results:**

Transcriptome analysis indicated that functions such as metabolism, digestion, and transport may become reduced due to the downregulated expression of many associated genes. During the molting stage, midguts harbor less microflora and DNA synthesis decreases. Both ecdysone and juvenile hormone in the larval midgut likely degrade after entering the larva-to-larva molting stage. However, at 12 h after ecdysis, the feeding larvae of 5th instars that were injected with 20-hydroxyecdysone entered a molting-like stage, during which changes in midgut morphology, DNA synthesis, gene expression, and microflora exhibited the same patterns as observed in the actual molting state.

**Conclusion:**

This study is important for understanding insect midgut physiology, development and immunity during a special development stage when no food is ingested. Although the molting larva becomes immobile and quiescent, we demonstrate that numerous changes occur in midgut morphology, physiology, metabolism and microbiome during this period.

**Electronic supplementary material:**

The online version of this article (doi:10.1186/s12864-016-3162-8) contains supplementary material, which is available to authorized users.

## Background

Insects are a model for studying human intestinal infection and pathology due to their similarities in signal transduction, gene regulation and cellular development [1, 2]. The insect gut is composed of the foregut, midgut, and hindgut [3]. In the foregut, food is mixed with enzymes derived from the foregut and salivary gland and are then transferred to the midgut, which is the primary location for food digestion and nutrient absorption [3]. Food debris is then transferred to the hindgut for water, salt, and some nutrient absorption [3]. In herbivorous insects such *Bombyx mori*, prophenoloxidase (PPO) produced by hindgut epidermal cells eliminates the majority of microorganisms in the food debris by inducing melanization of feces, thus avoiding the distribution of potential pathogens into the surrounding habitat [4]. In the insect foregut, there is also PPO that can detoxify food phenolics [5]. Aside from these physiological functions, recent attention has focused on development and microbiota interactions in the midgut of various species of insects [2, 3, 6]. In the midgut of *Drosophila melanogaster*, insulin signaling enhances intestinal stem cell division to drive tissue growth [7, 8]. When physical damage or pathogenic infection occurs in the midgut, intestinal stem cell proliferation is induced [9–11]. Several cell signaling pathways, such as JAK-STAT, decapentaplegic (DPP), and Wingless/Myc, affect intestinal stem cell division and differentiation in *Drosophila* [3]. Many microorganisms reside in the insect midgut; e.g., bacteria, fungi, and parasites [2, 3, 6]. Bacteria are the main microorganisms in the midgut of most insects, and they exhibit marked divergence depending on the host species, development, and location within the gut [2, 3, 6]. Commensal bacteria facilitate food digestion and can produce nutrients that are otherwise unavailable due to nutrient-poor diets [2, 6]. Some bacteria can also mediate host insect communication, reproduction, and pathogen exclusion [2, 6]. Thus, commensal bacteria in the midgut are important for insect development [2, 3, 6, 12].

Molting is a special period of the arthropod life cycle. During this time, organisms stop eating and become quiescent to prepare for shedding of the old cuticle [13]. In most insects, several molts occur during the entire life cycle. During each molting stage, molting fluids are produced and stored between the old and new cuticles [14]. During each molting period, a new cuticle is produced, and the transcription of several genes in the epidermis is upregulated [13, 14]. Thus, changes involving gene transcription, protein production, and tissue replacement in the epidermis of molting larvae occur while the larva appears quiescent.

Insect midguts experience significant changes in structure and function during metamorphosis to accomplish larva-to-pupa or pupa-to-adult ecdysis [3, 15–17]. In the Lepidoptera (e.g. *Bombyx mori*) and Diptera (e.g. *Drosophila melanogaster*), larval intestinal tissues degenerate through apoptosis and autophagy during the pupation period [16, 18]. When pupal midgut cells are produced from larval progenitors through cell division and differentiation, the original larval midguts are sequestered and form the component yellow body [15, 16]. In addition to these morphological changes, the microflora in the midgut also changes drastically during metamorphosis. In mosquitos, bacteria are abundant in the midgut during the fourth larval feeding stage but substantially decrease toward the pupal and adult stages [19]; this process appears to be a form of gut sterilization. The midgut morphology and microflora clearly undergo substantial changes during the metamorphosis stage. In Lepidoptera, prior to metamorphosis to become pupae and adults, insects experience several larva-to-larva molting periods [13, 14].

In the present study, we examined the *Bombyx mori* midgut during the fourth larva-to-larva molting stage to investigate whether molting midguts experience any previously unknown changes. The microarray assay indicated that many genes related to metabolism, transport, and proteolysis were downregulated, indicating that the corresponding physiological functions also likely decreased. Our data further indicate that the numbers of cultivatable bacteria in the midgut decreased rapidly during the molting stage, until very few bacteria were detected. In addition, DNA synthesis of midgut cells decreased during the molting stage. Increasing the titer of 20-hydroxyecdysone (20E) via injection into larvae induced changes in the number of cultivatable bacteria, DNA synthesis, and gene expression in the insect midguts, all of which were similar to the changes that occurred during the larva-to-larva molting stage. Additionally, the increased 20E titer in hemolymph likely induced the noted physiological changes within the midgut.

## Methods

### Insect feeding and dissection

Silkworm larvae (*Nistari*) were reared on mulberry leaves at 25 °C under a 12-h photoperiod. For *Nistari* used in this work, the 4th instar lasted 4 days, the molting to 5th instar lasted 18–20 h and the 5th instar lasted 5 days. We choose the end (at 12 h of day 3) of the 4th feeding stage, which is very near to a molting stage, and the time point immediately after ecdysis to compare structure and gene transcriptional changes in the midguts before and after the molting stage. The 4th molting stage is 18–20 h long. The midgut was sampled at different time points of this molting stage based on morphological and/or midgut content changes. Larvae from the following stages were used in the study: 1) from day 3 of the fourth feeding stage (IV-3:12 h), 2) a varying number of hours (X) after the beginning of the fourth larva-to-larva molting stage (IV-M: X h), 3) new larvae immediately after ecdysis (V-0:0 h), and 4) day 1 larvae of the fifth feeding stage (V-1:0 h). To obtain the midguts, silkworm larvae at different developmental stages were dissected in sterilized 0.85 % NaCl after bleeding. The dissected midguts were washed in 0.85 % NaCl five times to remove hemolymph.

### Oligonucleotide microarray

Several papers concerned with gene regulation of silkworm midgut development and immunity have been published using microarray assays [18, 20, 21]. In order to compare those data directly, transcription changes of the molting midgut were analyzed using the same method. Briefly, RNA isolation, amplification, labeling, hybridization, and microarray imaging and data analysis were performed according to previous descriptions [18]. Midguts of silkworm larvae from the fourth feeding stage (IV-3:12 h) and 3 h after the beginning of molting (IV-M:3 h) were dissected. Isolated midguts were then pulverized in liquid nitrogen and stored at −80 °C in TRIzol (Invitrogen, San Diego, USA). Total RNA (5 μg) was used to prepare fluorescent dye-labeled cDNA using a cRNA Amplification and Labeling Kit (CapitalBio) for the following microarray assay. Each group was replicated three times. Raw data were analyzed using BRB-Array Tools (http://linus.nci.nih.gov/BRB-ArrayTools.html). Expression data were normalized by centering each array using the median over the entire array as a reference. Genes with data missing from more than 25 % of samples were omitted, and a base-2 logarithmic transformation was applied to the dataset. Differentially expressed genes were selected using SAM [22] with a false discovery rate of 0.001. Significance was determined with q-values set at 1 % and an at least two-fold ratio of signal intensity between two developmental stages. Hierarchical clustering was performed using MultiExpromentViewer 4.9.0. Gene homology descriptions were provided by the array producer and were further confirmed by BLAST in GenBank. Gene ontology analysis was performed using the Molecular Homological Description System 4.0 (MAS V4.0, http://www.capitalbio.com) [23].

### 20-Hydroxyecdysone (20E) injection

The 5th instars were injected with 20E at several time points after ecdysis. Injects at 12 h post ecdysis (V-0:12 h) produced consistent results which injection at 24 h gave variable results. The callow larvae were fed for at least 12 h before inducing the molting stage. At 24 h after ecdysis, the efficiency of injected 20E to induce the molting state varied among individuals. Thus, we did not select this time point for 20E injection. Each silkworm larva (V-0:12 h) was injected with 10 μl of 20E (2 μg/μl; Santa Cruz, CA, USA) dissolved in 20 % dimethyl sulfoxide (DMSO). Larvae of the same age were injected with the same amount of DMSO as a control. At the scheduled time (24, 36 and 48 h after injection), silkworm larvae injected with 20E or DMSO as well as naïve larvae were dissected for their midguts, and RNA extraction and tissue sectioning were performed as described above.

### Quantitative RT-PCR (qRT-PCR)

The changes of some genes were analyzed during different molting and feeding stages and they appeared similar changes as 4th molting and 5th feeding stage (before wandering stage). Based upon the microarray results, we further analyzed via qRT-PCR genes expressed during the 4th feeding, 4th molting and 5th feeding stages. Total RNA was extracted from midguts at different developmental stages using the TRIzol reagent followed by treatment with RNase-free DNase I. mRNA from 3 μg of total RNA was transcribed into single-strand cDNAs using a first-strand cDNA synthesis kit (ToYoBo, Osaka, Japan) according to the manufacturer’s protocol. All specific primers were designed using the online Primer3 internet-based interface (http://biotools.umassmed.edu/bioapps/primer3_www.cgi) (listed in Additional file 1: Table S1). qRT-PCR reactions were performed in a 20-μl volume containing 10 μl of 2 × SYBR Green Master Mix (ToYoBo), 2 μl of cDNA, 1 μl of each primer (10 μM), and 6 μl of H_2_O. The PCR reaction was performed on a Bio-Rad CF × 96™ Real-time System using the following program: 95 °C for 3 min, followed by 39 cycles of 95 °C for 10 s, 55 °C for 30 s, and 72 °C for 10 s. Ribosomal protein S7 (rps 7) was used as an internal control. All samples were measured independently three times. Relative transcription abundances (2^-ΔΔCT^) were calculated using the equation of 2^-ΔCT^, where ΔCT was calculated as follows: CT target gene–CT rps 7.

### BrdU labeling and detection

Previous studies have used BrdU (5-bromo-2′-deoxyuridine) to detect proliferating cells [24]. Briefly, silkworm larvae at different developmental stages or at various times post 20E or DMSO injection were weighed, anesthetized on ice, and then separately injected with 0.5 mg/g body weight of BrdU (Invitrogen, San Diego, USA). The BrdU-labeled midguts were dissected 3 h later and then fixed in Bouin’s fluid for 12 h and stored in 70 % ethanol. The samples were then dehydrated through a series of graded ethanol baths to displace the water, and then infiltrated with paraffin wax followed by tissue section (5 μm). Slides containing samples were deparaffinized and rehydrated followed by incubation in 2 N HCl for 20 min at room temperature. After that, slides were washed in PBS buffer one time and incubated in 0.1 M Na_2_B_4_O_7_ for 10 min. After washing in PBS buffer three times, an anti-BrdU (IgG_1_) monoclonal antibody produced in mice (1:100; Invitrogen, San Diego, USA) was used as the primary antibody, and Rhodamine-conjugated goat anti-mouse IgG_1_ (1:100; Santa Cruz, CA, USA) was used as the secondary antibody to detect midgut cells that incorporated BrdU. DAPI (4′,6-diamidino-2-phenylindole) was used to stain nuclei. All images were taken using a fluorescence microscope (Olympus BX51).

### Histological staining

Insect midguts at different developmental stages or at various times post 20E or DMSO injection were fixed as described above. Tissue sections (5 μm) were stained with 2 % Mayer’s hematoxylin and 1 % eosin as described previously [25]. Slides were observed and photos were taken using a fluorescent microscope.

### Enzyme activity assay

To assay enzyme activities, midguts dissected from different developmental stages were sonicated in 10 mM Tris-HCl (pH 7.4) and centrifuged at 10,000 × g at 4 °C for 10 min. The total protein concentration of each supernatant was determined [26]. The supernatant (approximately 50 μg) was then used immediately. To obtain the midgut contents, the dissected midguts were washed in sterilized 0.85 % NaCl three times and then cut open to expose the peritrophic membranes that were then removed by nipping both ends using two forceps. The intact peritrophic membranes were also washed in 0.85 % NaCl three times and the surface solution was removed using absorbent clean paper. After that, the above dissected peritrophic membrane was cut open to release contents in 2 ml cold 0.85 % NaCl (4 °C). The midgut contents from three larvae were vortexed and centrifuged at 10,000 × g at 4 °C for 10 min to obtain the supernatant for enzyme activity assays. Methods to detect saccharase [27] and aminopeptidase N [28] activities were performed as described elsewhere. The activity of alkaline phosphatase was measured as described previously [29]. The absorbance of the supernatant was read at 550 nm and was used to determine alkaline phosphatase activity. To detect total protease activities in the native gel [30], the midgut contents (12 μl of suspended solution) from each developmental stage were loaded onto the gel. After separation, the gel was incubated in 50 mM Tris–HCl, pH 7.5, containing 10 mM CaCl_2_ at room temperature for 60 min before staining in Coomassie Brilliant Blue R-250 solution. After destaining, the gel bands containing proteases appear white in color.

### Cultivable bacteria in the midgut

Larval midguts from stages IV-3:12 h to V-1:0 h that included the different time points of the fourth molting stage were dissected. The midguts were washed using sterilized 0.85 % NaCl several times to remove hemolymph and were then cut open after drying for 5 min. All gut contents were removed and weighed. After suspension in sterilized 0.85 % NaCl solution in proportion (0.1 g wet weight to 2 ml solution), 50 μl were removed and streaked on a Luria-Bertani (LB) plate that was maintained at 37 °C for 15 h. Bacterial colonies were then counted.

### SDS-PAGE and Western blot analysis

The silkworm larvae midguts at different stages were dissected, homogenized and sonicated in 10 mM Tris-HCl (pH 7.4) containing 1 mM PMSF on ice, and centrifuged at 10,000 × g at 4 °C for 10 min to collect supernatants. Protein concentrations were determined [26]. Approximately 10 μg of protein was loaded per lane, separated by 12 % denaturing SDS-PAGE, and then transferred to a nitrocellulose membrane for a Western blotting assay. Antibodies against *Manduca sexta* βGRP2 [31] and Serpin-3 [32], *B. mori* ecdysone oxidase and 3-DE 3α-reductase [33], CPG21 (a gift from Dr. Ningjia He) and tublin (Vazyme, NanJing, China) were used as primary antibodies separately, and an AP-conjugated goat anti-rabbit IgG (1:5,000) or an HRP-conjugated goat anti-rabbit IgG (1:5,000) was used as the second antibody separately. Tubulin protein was used as loading control.

### LC-MS/MS

The samples of silkworm larvae midguts (IV-M:6 h) used for LC-MS/MS was prepared as above. Approximately 15 μg total proteins were loaded for denaturing SDS-PAGE separation followed by Coomassie Brilliant Blue R-250 staining. Based on the molecular sizes of cuticle proteins listed in Additional file 2: Table S2, the gels that appeared to contain cuticle proteins were excised for LC-MS/MS assay as previously described [4]. The two databases used in this study were SilkDB (http://silkworm.genomics.org.cn/) and the *B. mori* protein sequence database which was downloaded from NCBI (www.ncbi.nlm.nih.gov) using the keyword “*Bombyx mori*”.

## Results

### Functions of metabolism and transport are down-regulated in the molting midguts

**Fig. 1:**
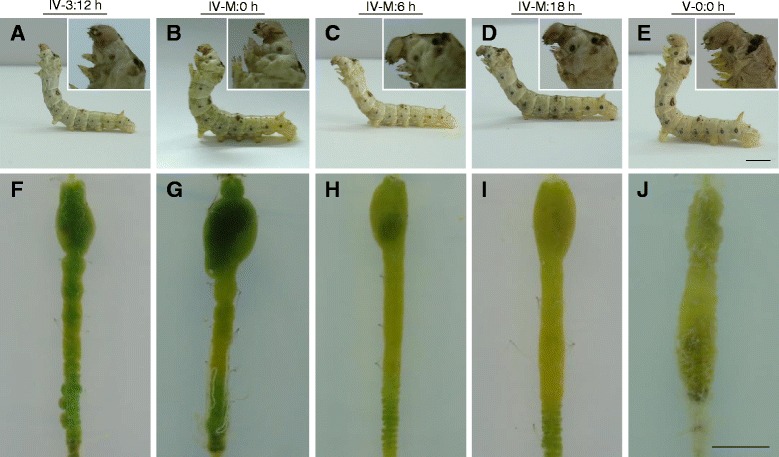
Morphological changes of silkworm larvae and midguts during the fourth larva-to-larva molting stage. **a**–**e** Changes in the appearance of larvae from day 3 of the fourth larval feeding stage (IV-3:12 h) to day 0 of the fifth larval stage (V-0:0 h). IV-M: X h indicates X hours after the beginning of the fourth molting stage (**b**–**d**). **e** The newly emerged larva (V-0:0 h) was imaged immediately after ecdysis. The *inset* in each photograph is an enlarged image of the head of the corresponding larva. **f**–**j** Morphology of the larval midgut at the indicated developmental stage. Food is present in the midguts of larvae at IV-3:12 h (**f**) and at the beginning of the molting stage (**g**). Very little food was observed in the midgut after the initiation of molting (**h**). No obvious food was found in the midguts of other stages (**i**–**j**). Obvious morphological changes were not observed during the molting stages. Bar: 5 mm

Lepidopteran insects, including the silkworm *Bombyx mori*, enter the larva-to-larva molting stage and shed the old larval cuticle periodically before pupation. During these larval molting stages, silkworm larvae become quiescent after affixing themselves to a supporting structure. In contrast to the feeding stage (Fig. 1a), the larval head becomes round during the molting stage (Fig. 1b–d). After shedding the old cuticle, the insect bodies are very soft and pale, but also larger (Fig. 1e). While appearing quiescent, changes in the integument are actively occurring [14]. Before, or at the very beginning of the molting stage, midguts were observed to be full of food (Fig. 1f-g). However, midguts dissected during the molting stage did not contain food; thus, it appeared that the remaining food was thoroughly digested into very small debris (Fig. 1h-j).

**Fig. 2:**
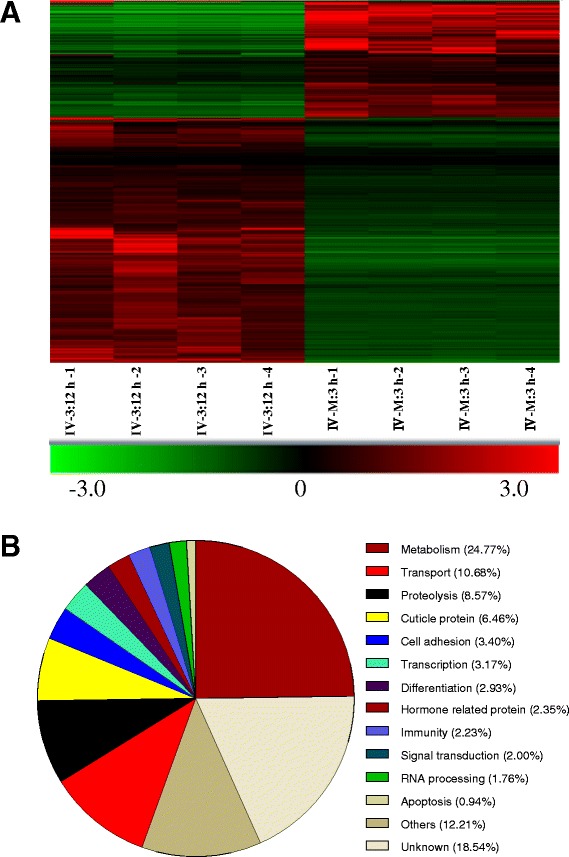
Transcriptome changes in the midguts at the feeding stage (IV-3:12 h) and at 3 h of the fourth larva-to-larva molting stage (IV-M: 3 h). **a** Heat map of the relative expression levels of genes as determined by a microarray assay. *Red and green* indicate higher (positive value) and lower (negative value) expression levels, respectively. **b** Gene ontology (GO) assignments for the transcriptome changes in the midgut at two different developmental stages. Predicted GO assignments for involvement in biological processes and molecular functions are indicated

The overall expression patterns of genes in the larval midgut were compared during feeding (IV-3:12 h) and molting (IV-M:3 h) stages (Fig. 2a; Additional file 2: Table S2). Based on the microarray assay, there were approximately 852 genes exhibiting different levels of expression. Four groups of genes (comprising almost 50 % of the genes detected), such as those involved in metabolism (24.22 %), transport (10.68 %), proteolysis (8.52 %), and cuticle proteins (6.46 %), exhibited changes in expression (Fig. 2b). In the metabolism group, the expression of 211 genes changed; these genes can be classified into those involved in the TCA cycle or the metabolism of carbohydrates, amino acids, nucleic acids and lipids, vitamins, chitin and others (Table 1). Approximately 78.2 % of metabolism-related genes were downregulated upon entry to the molting stage. In addition, there were over 70 % of genes in six groups (the metabolism of carbohydrates, amino acids, nucleic acids and lipids, TCA cycle and others) that had low transcription levels during the early molting stage. In the vitamin and chitin metabolism-related gene groups, there was a limited number of genes that had low transcription levels during the beginning of the molting stage. In the midguts of wandering larvae before the pupal molt, most of the metabolism related genes were down-regulated [18]. However, in the midguts of molting larvae, these metabolism-related genes (such as BGIBMGA003057-TA, BGIBMGA014149-TA, BGIBMGA014192-TA, BGIBMGA013860-TA and BGIBMGA005696-TA) were down-regulated during the early molting stage but then were up-regulated later. It appears that the larvae were ready for entering the next feeding stage after ecdysis. Thus, it appears that gene transcriptional changes are very different among wandering [18] and molting stages (this study) when larvae do not ingest food. Chitin synthase B had a low level of gene transcription during the early molting stage and then recovered to almost the same level as the feeding stage (IV-3:12 h). We conclude that this gene may synthesize chitin for the midgut since the new peritrophic membrane, which contains chitin, was produced during the molting stage (Fig. 7o). The group of transport-related genes (91) could be divided into those involved in the transport of amino acids and proteins, vitamins, ions, sugars, or neurotransmitters, and approximately 79.12 % of these genes were downregulated (Table 2). Seventy-three proteolysis-related genes exhibited transcription changes (Additional file 2: Table S2). Among these, approximately 78.08 % were downregulated. Only 6.46 % of cuticle proteins underwent transcription changes during the molting stage. The expression levels of genes involved in cell adhesion, transcription, differentiation, immunity, signal transduction, RNA processing, apoptosis, and hormones also changed (Fig. 2b); however, the levels of these groups were lower than the above four groups (metabolism, transport, proteolysis and cuticle proteins) of genes (Additional file 2: Table S2). In these microarray experiments, approximately 12.21 % of genes were classified into an “others” group. Approximately 19 % of genes with unknown functions also experienced changes in transcription during the molting stage. Midgut functions—such as metabolism and transport—likely decreased due to the downregulation of the expression of associated genes during the molting stage.

**Table 1:** ﻿Differentially expressed genes related to Metabolism

	Number of differentially expressed genes	Number of down-regulated genes (IV-M:3 h vs. IV-3:12 h) (%)
Metabolism^a^	211	165 (78.20 %)
Carbohydrate metabolism	51	40 (78.43 %)
Amino acid metabolism	31	26 (83.87 %)
Nucleic acid metabolism	12	11 (91.67 %)
Lipid metabolism	41	31 (75.61 %)
Vitamin metabolism	5	3 (60.00 %)
TCA cycle	10	9 (90.00 %)
Chitin metabolic process	11	4 (36.36 %)
Others	50	41 (82.00 %)

^a^The total number of all genes related with metabolism that had transcription changed

**Table 2:** Differentially expressed genes related to Transport

	Number of differentially expressed genes	Number of down-regulated genes (IV-M:3 h vs. IV-3:12 h) (%)
Transport^a^	91	72 (79.12 %)
Amino-acid and protein transport	10	9 (90.00 %)
Vitamin transport	6	4 (66.67 %)
Ion transport	29	23 (79.31 %)
Sugar transport	8	6 (76.67 %)
Neurotransmitter transport	5	4 (80.00 %)
Others	33	26 (78.79 %)

^a^The total number of all genes related with transport that had transcription changed

### Digestive enzyme activities decrease but do not disappear

**Fig. 3:**
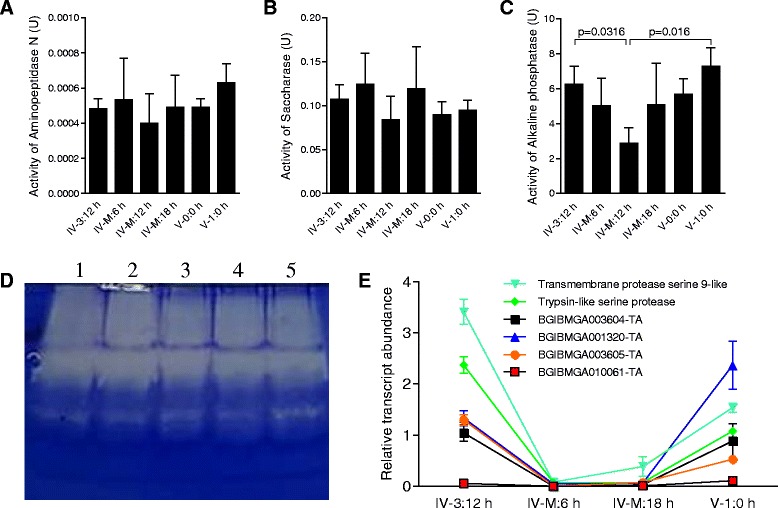
Digestive enzymes in the molting midguts and contents. The supernatants of cell lysates from midguts at different developmental stages were subjected to analysis for aminopeptidase N (**a**), saccharase (**b**), and alkaline phosphatase (**c**) activities, respectively. For each assay, approximately 50 μg of total protein were utilized. Activities of each enzyme were decreased toward the middle time point (IV-M: 12 h) of the molting stage and then enzyme activities subsequently increased. The activity of alkaline phosphatase was significantly lower at 12 h post molting than at other time points. **d** Protease activities in the midgut contents were detected using a native gel as described [30]. Lane 1 to Lane 5 are midgut contents from larvae at IV-3:12 h, IV-M: 0 h, IV-M: 12 h, IV-M: 18 h and V-1:0 h stages, respectively. For each lane, approximately 12 μl of midgut contents after suspension and centrifugation were loaded. **e** Transcription of genes encoding proteases in the midguts of larvae at different developmental stages were assayed by qRT-PCR. The transcription of these proteases was down-regulated to a very low level during the molting stage. During feeding stages, these genes were expressed at high levels. Each column represents the mean of three independent measurements  ±  S.E.M

*B. mori* insects experience wandering (larvae leave food), quiescent (larvae stop moving) and ecdysis (old cuticle is shed) stages during each larval molt. Lepidopteran larval midguts secrete many enzymes required for food digestion [12]. During each molting stage, larvae did not consume food, which caused us to investigate digestive enzyme activities in both the midgut and gut contents. There was no obvious change in saccharase at the transcription level. Since saccharase is a very important metabolic enzyme, we investigated whether it disappears during the molting stage. The activities of aminopeptidase N (Fig. 3a), saccharase (Fig. 3b), and alkaline phosphatase (Fig. 3c) all decreased within 12 h after the beginning of the molting stage and they all reached their lowest level at 12 h after the start of the molting stage. After that, the enzyme activities increased likely in order to become ready for ingesting food after ecdysis. Total protease activities in the midgut contents of molting larvae also were similar to those of feeding larvae (Fig. 3d). Several genes related to protein digestion (mainly trypsin) were subjected to additional transcription analysis. All such genes exhibited extremely low levels of transcription from the beginning to the end of the molting stage (Fig. 3e). All of these genes also exhibited high levels of transcription during the feeding stage. Between 6 and 12 h after the beginning of the molting stage, their transcriptional changes were all at the lowest level. These findings indicate that many enzymes are present in the midgut and its contents. The expression of some genes (Trypsin-like serine protease, BGIBMGA001320-TA, BGIBMGA003605-TA and BGIBMGA010023-TA) was completely or partially downregulated upon entering the molting stage. In the beginning of the molting stage, there was some larger food debris left in the midgut (Fig. 1g-h). After that, only small food debris was observed (Fig. 1i-j). Thus, it is not surprising to detect the presence of some enzymes to digest the remaining food debris in the molting midguts (see Fig. 1g). However, activities of these enzymes were decreased at the middle of the molting stage. These data suggest that the molting larvae may obtain energy through digesting the remained food in the molting midgut until the food become small debris observed in the late molting stage (Fig. 1i-j). Obviously, the above enzymes detected in the molting midgut contents are enough to digest the any remaining food.

### Metabolism of hormones in the molting midguts

**Fig. 4:**
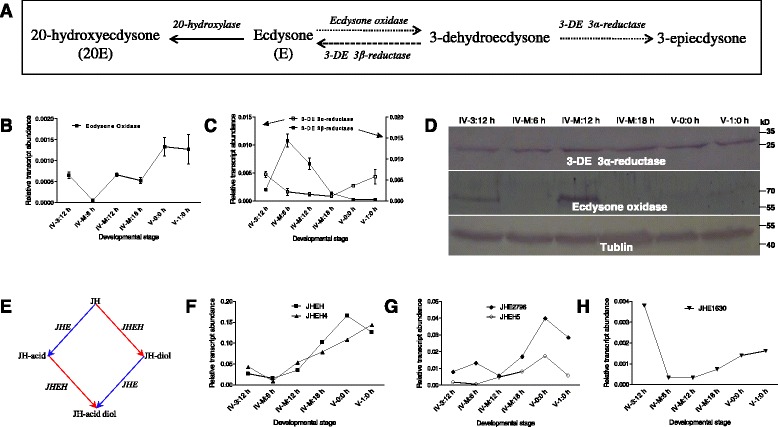
Hormone-degrading enzymes in the midguts. **a** In the ecdysone metabolic pathway in insects [35, 60], ecdysone that is released from the prothoracic gland cells is either hydroxylated into 20E or metabolized into different intermediate products after oxidization in different tissues. **b**–**c** Expression of genes related to ecdysone degradation in the midgut from IV-3:12 h to V-1:0 h. The changes in expression of these genes differed between the two feeding stages according to qRT-PCR assay results. **d** Detection of ecdysone oxidase and 3-DE 3α-reductase in midguts by Western blot. Antibodies against *B. mori* ecdysone oxidase and 3-DE 3α-reductase were used for the Western blot assay [33]. Samples are midguts of larvae from IV-3:12 h to V-1:0 h. For each lane, approximately 10 μg was loaded. 3-DE 3α-reductase was observed at each time point. However, large amounts of ecdysone oxidase appeared at the middle time point. **e** The JH metabolic pathway in insects is based on previous descriptions [41]. **f**–**h** Expression of genes involved in JH metabolism in the midgut were assayed by qRT-PCR. These genes were identified in the microarray assay as described above (Fig. 2). These genes were generally up-regulated from the 4th molting stage to the feeding stage as assayed. Each column represents the mean of three independent measurements  ±  S.E.M

In this microarray assay, approximately 2.35 % of genes were hormone-related. Most of these genes exhibited a close relationship with ecdysteroids or juvenile hormone (JH) metabolism (Additional file 2: Table S2). During the larval feeding stage, the titer of plasma ecdysteroids periodically increases towards the beginning of each larva-to-larva molting stage and then decreases to a very low level after ecdysis [34]. Ecdysone oxidase, 3-DE 3α-reductase, and 3-DE 3β-reductase catalyze the metabolism of ecdysone [35] (Fig. 4a) and ecdysone is converted into 20E by 20-hydroxylase in many tissues [36]. It is 20E, which initiates molting and metamorphosis in insects [34, 37]. The midgut also contains both ecdysone oxidase and 3-DE 3α-reductase simultaneously, thus facilitating 20E metabolism to 3-epiecdysone in this tissue [36].

Transcription of the gene encoding ecdysone oxidase differed markedly between the feeding and molting stages. Ecdysone oxidase gene transcription decreased from the IV-3:12 h feeding stage to the early molting stage (IV-M:6 h) and then increased toward the V-1:0 h feeding stage (Fig. 4b). 3-DE 3α-reductase and 3-DE 3β-reductase in the midgut exhibited opposite responses (Fig. 4c). 3-DE 3α-reductase remained at lower levels during the molting stage but increased during all feeding stages (Fig. 4c). In contrast, the gene encoding 3-DE 3β-reductase exhibited high levels of expression during the molting stage (Fig. 4c).

Ecdysone oxidase and 3-DE 3α-reductase are detected in Lepidopteran insect midguts on a protein level [33]. Using the appropriate antibodies, 3-DE 3α-reductase was clearly detected in the molting midguts at different time points (Fig. 4d). For ecdysone oxidase, there was a large amount of it present in the middle period of the molting stage compared with other time points (Fig. 4d). Additionally, during the early and later molting stages, small amounts of ecdysone oxidase could be detected if the loading amounts were increased (data not shown). After the 4th ecdysis, ecdysone oxidase was clearly detected even though it was still in low amounts. Although this study did not assess the protein level of 3-DE 3β-reductase, a previous study has indicated that there is very low amounts of it in the *B. mori* midgut [38]. Thus, ecdysone oxidase and 3-DE 3α-reductase probably lead to ecdysone degradation in the molting midgut. Ecdysone likely remains at high levels in the midgut at the beginning of the molting stage due to small amounts of ecdysone oxidase.

Prior to metamorphosis, the hemolymph JH titer is high, although it decreases slightly after each larval-larval ecdysis [34, 39, 40], which differs from the hemolymph ecdysteroids titer. Juvenile hormone epoxide hydrolase (JHEH) and juvenile hormone esterase (JHE) can directly degrade JH into different products [41] (Fig. 4e). Several genes related to JH degradation were downregulated during the early molting stage (Additional file 2: Table S2). In terms of expression changes from IV-3:12 h to V-1:0 h, JHEH, JHEH4, JHEH5, JHE1630, and JHE2796 all increased during the molting stage (Fig. 4f–h). Each of these genes, with the exception of JHE1630, exhibited higher expression, even after ecdysis. Thus, the JH titer in the midgut probably remains high at the beginning of the molting stage. Toward the end of the molting stage, the JH titer decreased when JHE and JHEH were upregulated. These data indicate that the 20E and JH titers remain high at the beginning of larva-to-larva ecdysis and are then degraded in the molting midgut as development progresses.

### DNA synthesis is 20E dependent in the molting midguts

**Fig. 5:**
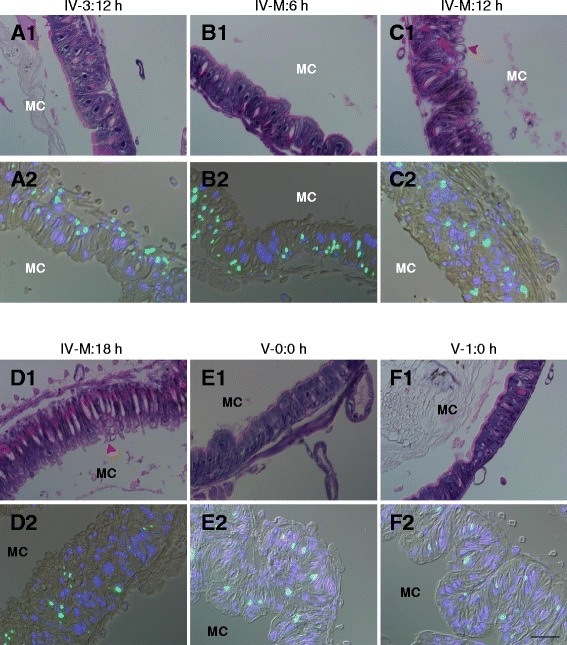
Morphological changes and DNA synthesis in the molting midguts. (*A1*–*F1*) Morphology of midguts at feeding stages (*A1*, *E1*, and *F1*) and the molting stages (*B1*–*D1*) observed by microscopy. The tissue sections were stained using Mayer’s hematoxylin and eosin. Bubble-like structures appeared during the molting stage (indicated by *arrowheads*). No such structures were detected during the feeding stages. (*A2*–*F2*) Decreased DNA synthesis was noted in the midgut after entering the fourth larva-to-larva molting stage. DNA synthesis indicates midgut growth even during the molting stage. Developmental stages are indicated. MC: midgut contents. Bar: 50 μm

**Fig. 6:**
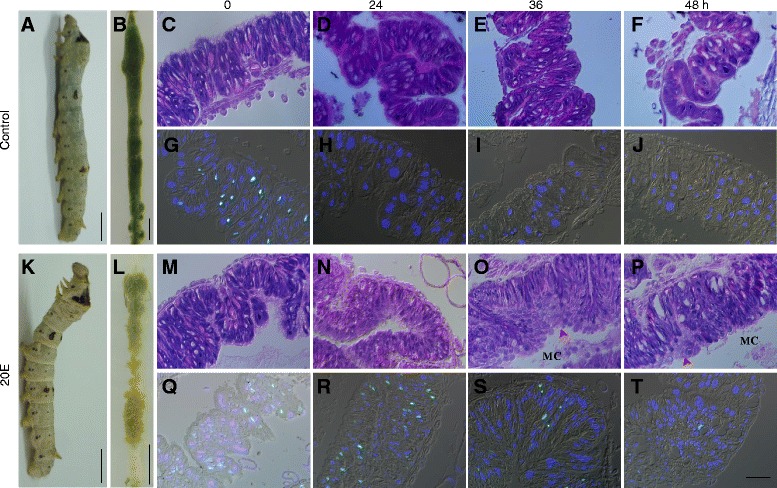
20E injection induces DNA synthesis in the midguts. **a**–**b** Appearance of a larva (**a**) and dissected midgut (**b**) at 36 h post 4th ecdysis. The larva ingested food and the midgut was full of food. **c**-**j** Morphology of midguts and DNA synthesis at different time points of naïve larvae as observed by microscopy. 20E was injected at 12 h post 4th ecdysis (see next) and is labeled as 0 h. Correspondingly, at each time point indicated, microscopic morphology changes (**c**-**f**) and DNA synthesis (**g**-**j**) in naïve larvae were observed. BrdU was incorporated into midgut cells at 0 h (12 h post ecdysis) (**g**). After this time point, no cell incorporated BrdU. **k**-**t** Microscopic morphology of midguts and DNA synthesis at different time points after 20E injection. At 12 h after the fourth ecdysis, 20 μg of 20E was injected into each larva. **k**-**l** Appearance of a larva (**k**) and dissected midgut (**l**) at 24 h post 20E injection (equal to 36 h post ecdysis). The insect appeared to be in a molting state at 24 h post 20E injection (**k**), and the midgut was devoid of contents (**l**). Morphological changes (**m**–**p**) and DNA synthesis (**q**–**t**) were compared. Bubble-like structures (indicated by *arrowheads*) were produced at 36–48 h post injection of 20E (**c**–**p**). DNA synthesis was induced and also decreased over time (**q**–**t**). Bars: 5 mm (**a**, **b**, **k**, **l**) and 50 μm (**c**–**j**, **m**–**t**)

Morphological changes and cell division in the midgut were monitored during the various molting stages. According to midgut morphological changes (Fig. 1f-j) observed after tissue sectioning with hematoxylin and eosin staining (Fig. 3A1–E1), the midgut may not grow quickly during the molting stage. However, after ingesting food, the midguts grew large from V-0:0 h to V-1:0 h (Fig. 1j). Bubble-shaped materials were observed in many locations adjacent to gut contents in the molting midguts (Fig. 5C1–D1). No such structures were observed in midguts during the feeding stages or the early molting stage (Fig. 5A1, B1, E1, and F1). The structure of the bubble-like materials is unknown. Although approximately 1 % of apoptotic genes exhibit changed transcription (Fig. 2 and Additional file 2: Table S2), no obvious apoptosis occurred in the molting midguts (data not shown). However, a previous study has shown that casual apoptotic cells were engulfed by neighboring cells rapidly [42]. We thus conclude that casual apoptotic cells were probably cleared quickly in the midguts during the molting stage. BrdU incorporation into midgut cells decreased gradually from the IV-3:12 h to V-1:0 h stages (Fig. 5A2–F2). The data indicate that DNA synthesis occurs throughout the molting stage and into the next instar (Fig. 5A2 and B2). The amount of DNA synthesis appears to decrease during the molt but quantification of BrdU incorporation was not possible for technical reasons. BrdU incorporation seldom occurred after the V-1:0 h stage (see Fig. 6h). DNA synthesis in cells indicated that the midguts still grow during the feeding stage. We did not detect signal, if BrdU was not injected or the primary antibody was omitted (Additional 3: Figure S1). The changes in DNA synthesis in the molting midguts were very similar to the change of 20E titer [34, 43], which indicated that midgut cell DNA synthesis has a close relationship with 20E.

When larvae at 12 h after the fourth ecdysis (V-0:12 h stage) were injected with 20E, they entered a molt-like state after 24–36 h (Fig. 6k). Morphological changes occurred in the midguts of larvae that received 20E injections. Larvae also stopped eating and became quiescent and the midguts were devoid of food (Fig. 6l). The midgut exhibited bubble-like structures in some locations (Fig. 6n–p), similar to the midgut at the fourth molting stage. DMSO (solvent for 20E) injection had no effect on the morphological changes of midguts (data not shown). 20E injection induced continuous DNA synthesis despite the decreased number of BrdU-labeled cells over time (Fig. 6q–t), which is also similar to that during the molting stage. In the control (naïve larvae), DNA synthesis occurred only at the beginning of the 5th larval stage (Fig. 6g) and DMSO did not affect BrdU incorporation as compared with naïve larvae (Additional file 3: Figure S1). These data indicate that 20E injection resulted in DNA synthesis in a few midgut cells in vivo, which is consistent with the observation in vitro [44, 45].

### Cuticle related proteins are involved in the development of molting midguts

**Fig. 7:**
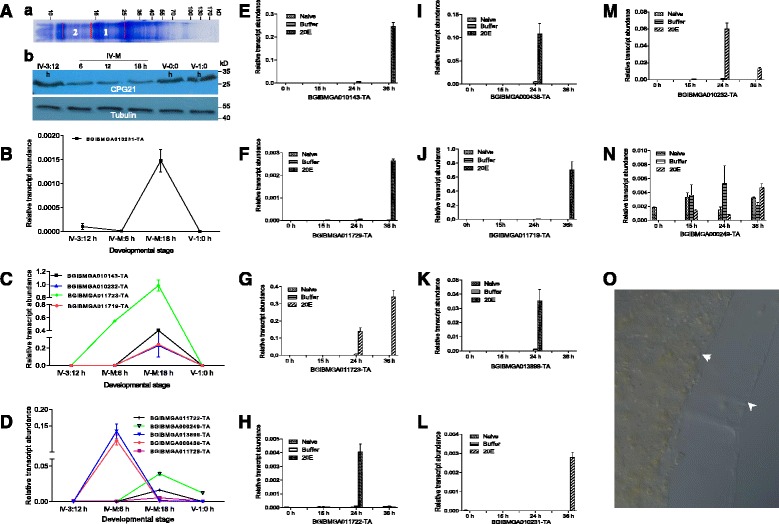
Regulation of cuticle protein expression in the molting midguts by 20E. (**a**) Detection of cuticle protein in the molting midguts. (**a**) Molting midguts dissected from three larvae (IV-M: 6 h) were lysed and 15 μg total proteins were loaded per lane for Coomassie staining. Two bands that likely contained different cuticle proteins according to their molecular weight sizes were excised for a LC-MS/MS assay. (b) Detection of CPG21 in the molting midguts by Western blot. CPG21 appeared during the feeding and molting stage. Unfortunately, there was no corresponding probe for CPG21 in the microarray. Thus, we could not assay for its transcription change. **b**–**d** Expression of selected cuticle genes at the indicated developmental stages according to qRT-PCR assay. These genes were selected according to the microarray assay (Fig. 2, Additional file 2: Table S2). The expression levels of these genes were upregulated at different times during the molting stage. **e**–**n** 20E injection induced the expression of selected cuticle genes in the midgut according to qRT-PCR assay. Among 10 selected genes, only one gene (BGIBMGA000249-TA; **m**) did not exhibit a clear response to 20E injection. Each column represents the mean of three independent measurements  ±  S.E.M. **o** Production of new peritrophic membrane during the molting stage. A new peritrophic membrane was produced and covered the old one. The *arrowhead* indicates the newly produced peritrophic membrane, and the *arrow* indicates the old peritrophic membrane and enclosed food contents. Food debris was small and visible in the gut. The peritrophic membrane is composed of many cuticle proteins [47, 48] that are likely produced by midgut cells during the molting stage

In the silkworm, the knock-down of CPH45 cuticle protein induced the larval midgut into an abnormal state after ecdysis [46], which indicates that cuticle proteins are important for midgut development. During the molting stage, 6.46 % of genes encoding cuticle proteins exhibited upregulated transcription (Additional file 2: Table S2; Fig. 2b). After midgut lysates were separated by denatured SDS-PAGE and subjected to a LC-MS/MS assay; several cuticle proteins (BGIBMGA002548-TA, BGIBMGA000324-TA, BGIBMGA010143-TA and CGP25) were identified (Fig. 7a; Additional file 4: Table S3). A Western blot also showed that there was CPG21 in the molting midguts (Fig. 7a). Several cuticle-related genes were subjected to transcriptional assays from stage IV-3:12 h to V-1:0 h, with most of these genes expressed during the late molting stage (Fig. 7b–d). While the expression of some of these genes was highest during the early molting stage (Fig. 7d), no transcriptional changes were observed during the fourth and fifth feeding stages (Fig. 7b–c). Following 20E injection, all genes with the exception of BGIBMGA000246-TA (Fig. 7n) exhibited marked transcriptional changes between 24 and 48 h post 20E injection (Fig. 7e–m).

In the peritrophic membrane of silkworm larvae, there are also cuticle proteins [47, 48]. For example, BGIBMGA010231-TA (putative cuticle protein) found in the peritrophic membrane [48] was regulated in the midgut during the molting stage (Additional file 2: Table S2). In the molting midgut, the new peritrophic membrane was often observed to cover the old one (Fig. 7o). We conclude that cuticle proteins are involved in midgut development and the remodeling of the peritrophic membrane during the molting stage.

### Reduction of microflora in the molting midgut after 20E increases

**Fig. 8:**
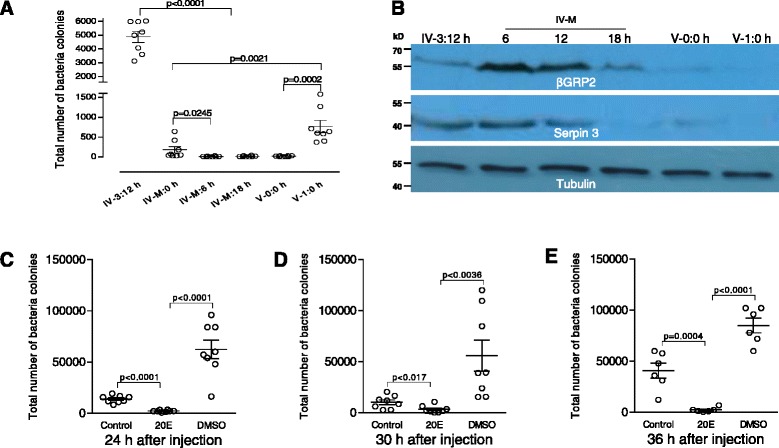
20E reduces the midgut microflora abundance likely by up-regulating the expression of immunity proteins. **a** Cultivable microflora in the midguts from IV-3:12 h to V-1:0 h. The total number of bacterial colonies significantly decreased when larvae entered the fourth molting stage. The number of midgut bacteria increased again after food was provided. **b** Expression of immunity proteins in the midguts during the molting stage as assayed by Western blot. Two antibodies against *Manduca sexta* βGRP2 [31] and Serpin-3 [32] were used for detection of the corresponding proteins in the molting midguts. These two proteins were observed to increase when either approaching or during the molting stage. **c**–**e** 20E was injected as described in methods. At 24–36 h post 20E injection, when the insects appeared to be in a molting state, midgut contents were removed for enumeration of cultivable bacteria. DMSO (solvent for 20E) injection was used as a control. 20E injection significantly decreased the abundance of the microflora within the midgut contents. Columns represent the means of independent measurements ± SEM (*n* = 8)

Before entering the molting stage, the cultivatable bacteria decreased significantly from IV-3:12 h (feeding) to IV-M:0 h (the beginning of molting) (Fig. 8a). The data indicate that the decrease occurred before the start of the molt (Fig. 8a). During the feeding stage, there were on average approximately 4000 CFU of cultivable bacteria. After shedding the old cuticle to become fifth-stage larvae, the number of midgut bacteria did not increase until food was provided after ecdysis to the 5th instar. Thereafter, the number of bacteria significantly increased within a day (Fig. 8a). A previous study [18] has shown that before pupation, some immunity related proteins were transcriptionally expressed in the silkworm midgut after the level of 20E increased, which likely clears the gut of microflora to help ensure safe metamorphosis. During the molting stage, the transcription of many immunity related genes was up-regulated (Fig. 2b; Additional file 2: Table S2) while the increased expression of some immunity proteins, such as βGRP-2 and serpin-3, was also noted (Fig. 8a). In insects, βGRP proteins can detect fungi and activate the Toll pathway [49] and serpins have been shown to be key regulators of innate immune reactions like melanotic encapsulation and Toll pathway activation [50–52]. The increasing immunity activity in the molting midguts may help to remove the midgut microflora. In insects, 20E has already increased before entering the molting stage [34, 43]. In *Drosophila*, the application of 20E also enhances antibacterial peptide production [53, 54]. We therefore hypothesized that the reduction in midgut flora was prompted, at least in part, by immunity proteins induced by the increase in 20E.

To test this hypothesis, 20E was injected into larvae at 12 h after the 4th ecdysis (V-0:12 h). During this period, the midgut contents were removed and cultivatable bacteria were enumerated. The midguts of larvae that received 20E injection contained significantly lower microflora abundances than those of control larvae or larvae that received only a DMSO injection (Fig. 8c-e). Surprisingly, DMSO injection increased the number of midgut bacteria; however, the underlying mechanism is unknown. When 20E or DMSO was co-cultured with *Escherichia coli* or *Bacillus subtilis*, neither significantly inhibited bacterial growth (data not shown). The microflora in feeding larval midguts of silkworms have been studied extensively, and several genera of bacteria were identified that were primarily *Micrococcus*, *Bacillus* and *Corynebacterium* [46, 47]. In the midguts of molting larvae in this study, the cultivatable bacteria were mainly *Escherichia coli* and *Staphylococcus aureus,* even though the number was very low (data not shown). Thus, it appears that the increasing 20E titer in the molting larvae induces immunity protein expression according to the changes in wandering larvae [18], which, in turn, results in a reduction in the midgut microflora.

## Discussion

Ecdysis is a crucial process for insect development. During their life cycle, holometabolous insects experience several cycles of ecdysis, which can be classified as larva-to-larva, larva-to-pupa, and pupa-to-adult moltings [14]. During each molting stage, insects stop feeding to prepare for entry into the next developmental stage. Although there is considerable information known about the larva-to-pupa and pupa-to-adult molts during metamorphosis [15], much less is known about the larva-to-larva molts. During each larva-to-larva molting stage, molting fluids accumulate between the old and new cuticles, which protect the larva within and ensure successful ecdysis [14]. Despite the outward quiescent appearance, many physiological functions of the insect midgut are changing during this time. Some digestive enzymes remain active in the midgut tissues and midgut contents in the molting larvae (Fig. 3d). Thus, food debris in the midgut is continuously digested to very small pieces after the initiation of molting (Fig. 7o). Surprisingly, many genes related to metabolism, proteolysis, and transport were downregulated, indicating that primary physiological functions, such as digestion and absorption, in the midgut appear to temporarily decrease during the larva-to-larva molting stage. Interestingly, the gut microflora was reduced, and very few cultivatable bacteria were detected during the molting stage (Fig. 8a). The abundance of midgut microflora did not increase until the new larvae began to consume food, indicating that the silkworm midgut microflora likely originates from the environment via food ingestion and/or from the proliferation of remained bacteria. In the bean bug *Riptortus pedestris*, the number of symbiont *Burkholderia* in the midgut decreased during the pre-molting stage, likely due to upregulation of antimicrobial activity [55] and at 24 h after oral infection of *B. mori* with *Bacillus bombysepticus* (*Bb*), many genes concerned with immunity and metabolism were significantly changed During the wandering stage of a previous study [18] and the molting stage of this study, there were some immunity related genes that were up-regulated in the midguts. More specifically, our data also suggested that the up-regulation of immunity proteins in the molting midguts reduced the midgut microflora (Figs. 2b, 8b; Additional file 2: Table S2). We therefore hypothesize that the upregulation of immunity likely serves to reduce the midgut microbiome during these two special developmental stages of wandering and molting. In this study, we found that there were obvious changes in metabolism related genes, such as beta-glucosidase, transport proteins (solute carrier families and putative inorganic phosphate co-transporter), protein digestion related proteins (aminopeptidase N and carboxypeptidase B precursor) and low molecular mass lipoprotein, in the feeding and molting stages.

Several cuticle proteins were detected in the molting midgut (Additional file 2: Table S2). After the midgut lysates were separated by denaturing SDS-PAGE and subjected to a LC-MS/MS assay; several cuticle proteins were identified (Fig. 7a; Additional file 4: Table S3). In a previous study, when the CPH45 cuticle protein was knocked-down in the silkworm, the larval midgut became abnormal after ecdysis, and the resistance of the insects to cytoplasmic polyhedrosis virus (CPV) decreased [46]. Together, these findings indicate that the insect midgut contains cuticle proteins whose expression levels change during the larva-to-larva molting stage (Fig. 7, Additional file 2: Table S2).

In the molting midguts, there also were approximately 1 % of apoptotic genes that exhibited different levels of expression (Fig. 2; Additional file 2: Table S2). Apoptosis occurs normally during development [56] and it has been previously shown that casual apoptotic cells are rapidly engulfed by neighboring cells [42]. Thus, we conclude that the casual apoptotic cells are likely cleared quickly in the midguts during the molting stage, thereby making it difficult to detect such cells in the midguts during this special stage (Fig. 2b).

*Nistari* larvae used in this study have almost the same length of 4th stage (feeding and molting) as the European breed 200 × 300 [57]. According to the published data [57], the titer of ecdysone was probably increased to 275 ± ng/ml at 12 h on the 3rd day (IV-3:12 h) and reached the peak (550 ng/ml) at the initiation of molting (IV-M:0 h). The insect midgut is a hormone-sensitive organ. Many larval tissues, including the midgut, are capable of converting ecdysone to 20E [58, 59]. Hemolymph 20E peaks at the initiation of the molting stage and decreases gradually thereafter [34, 43]. Ecdysteroids stimulates proliferation and differentiation of midgut stem cells in a concentration-dependent way [44, 45]. Ecdysone and 20E were particularly active in midgut cell proliferation and differentiation, respectively. We believe that there is likely a proper ratio of ecdysone and 20E in the midgut to balance cell proliferation and differentiation in this tissue. Large amounts of ecdysone oxidase in the middle time period of the molting stage should lead to ecdysone decrease in the midgut. Ecdysone enhances midgut cell proliferation in a dose responsive manner [45]. However, when the ecdysone titer is at low level, tissue damage or pathogenic infection induces the insect midgut stem cells into proliferation [9–11]. Physical damage to the midgut can induce DNA synthesis within a limited time (approximately 48 h) and space (around the wound) [58]. We think that DNA synthesis of midgut cells is primarily driven by the change in ecdysteroids and not by the expression of apoptotic genes during the molting stage. It is necessary to identify which type of midgut cells can incorporate DNA. In the silkworm, there are no protein markers for columnar cells and goblet cells, respectively. We tried the tissue culture protocol developed by Smagghe et al. [44, 45] and found that the columnar cells and goblet cells in the silkworm larval midguts were very easily broken after being cultured for a while. Thus, it is difficult to differentiate cell proliferation and differentiation in the molting midguts when specific markers to distinguish different midgut cell types are not available.

Previous studies have shown that during each larva-to-larva molting stage, the JH level decreases but still remains high [34, 39, 40]. JH does not decrease to its lowest level until insects are ready to enter the pupa or adult stage [39]. In this study, several genes related to JH degradation were upregulated after entering the molting stage (Fig. 4f–g). And 20E injection at 12 h after ecdysis caused insects to re-enter a molting-like stage (Fig. 6k). Subsequently, midgut morphology, DNA synthesis, and the expression of several genes underwent changes similar to those observed during the actual molting stage (Figs. 6m–p, q–t, and 7). Meanwhile, the abundance of the midgut microflora decreased significantly when compared with naïve larvae and those that received a DMSO injection (Fig. 8c-e). The expression of osiris genes also was upregulated during the fourth molting stage (Additional file 2: Table S2). The functions of osiris genes are unclear; however, all osiris-related genes exhibited the highest level of expression during the early molting stage (Additional file 5: Figure S2A–B). Each of these genes was upregulated at approximately 18–24 h post 20E injection (Additional file 5: Figure S2C–J). JH application did not induce a molting state (data not shown). These results indicate that the larval midguts respond to 20E injection by exhibiting a molting-like state. However, molting midguts are not quiescent as the larvae appear. The changes in ecdysteroids appear to regulate larval development so that the larvae can prepare for their next feeding.

## Conclusions

Larva-to-larva molting in insects is a special period during which the old cuticle is shed and a new cuticle is produced [13, 14]. During this process, insect larvae remain restful and do not consume food. This raises the question of whether the midgut is also quiescent during the larva-to-larva molting stage. Our results indicate the presence of various enzymes in midgut tissue and midgut contents during this stage. DNA synthesis in midgut cells continues, although levels decrease before ecdysis. During the molting stage, the midgut microflora decreased significantly and even disappears in some molting larvae. The reason for such changes is probably to decrease the midgut physiological functions via the downregulation of genes involved in metabolism and transport. The increased 20E levels in hemolymph may be the primary factor to induce the changes in the molting midguts. However, further works are necessary to understand the physiological changes of molting midguts in the future.

## Additional files

Additional file 1: Table S1. Primers used for real-time PCR assay. (XLS 27 kb)
Additional file 2: Table S2. Genes identified by microarray analysis. (XLS 205 kb)
Additional file 3: Figure S1. DMSO did not increase BrdU incorporation according to observation by microscopy. DMSO was the solvent for 20E. As a control, the same volume of DMSO (20 %) was injected and the midguts were sampled. As another control, naïve and DMSO-injected larvae were also sampled at 0 h post injection. Some samples of naïve larvae (without 20E injection) and DMSO-injected larvae were stained with secondary (2nd) antibodies only (A-B). DMSO did not enhance BrdU incorporation (E-L). Without BrdU injection or by omitting the primary (1st) antibody against BrdU, there was no signal (A-D). Bar: 50 μm. (PPT 3272 kb)
Additional file 4: Table S3. List of cuticle proteins in the midguts of the molting stage after identification by LC-MS/MS. The gel bands were excised as shown in Fig. 7A-a. (PPT 106 kb)
Additional file 5: Figure S2. 20E increased the expression of osiris genes in the midgut. 20E injection was performed as described in methods. (A–B) Expression of osiris genes during the indicated developmental stages as assayed by qRT-PCR. Osiris genes assayed here were up-regulated at the early molting stage (6 h after the initiation of molting). (C–J) 20E injection induced the expression of osiris genes in the midgut. All osiris genes were up-regulated at 24 h post 20E injection. The changes were not obvious at other time points except Osiris 9-4 at 15 h post 20E injection. (PPT 330 kb)

## Abbreviations

20E: 20-hydroxyecdysone
BrdU: 5-Bromo-2′-deoxyUridine
CPV: Cytoplasmic polyhedrosis virus
DMSO: Dimethyl sulfoxide
IV-3:12 h: Day 3 of 4th larval feeding stage
IV-M: X h: X hours after the beginning of 4th molting stage
JH: Juvenile hormone
JHE: Juvenile hormone esterase
JHEH: Juvenile hormone epoxide hydrolase
LB: Luria-Bertani
LC-MS: Liquid chromatography–mass spectrometry
MC: Midgut content
PPO: Prophenoloxidase
qRT-PCR: Quantitative RT-PCR
rps 7: Ribosomal protein S7
SDS-PAGE: Sodium dodecyl sulfate polyacrylamide gel electrophoresis
TCA: Tricarboxylic acid cycle
V-0:0 h: Day 0 of 5th larval stage
V-1:0 h: Day 1 of 5th larval feeding stage
