# Supplementary figures and images for: Analysis of gene expression in the midgut of Bombyx mori during the larval molting stage
Source: BMC Genomics. 2016 Nov 3;17:866. doi: 10.1186/s12864-016-3162-8 (PMC5096333; doi:10.1186/s12864-016-3162-8)

## Slide 1
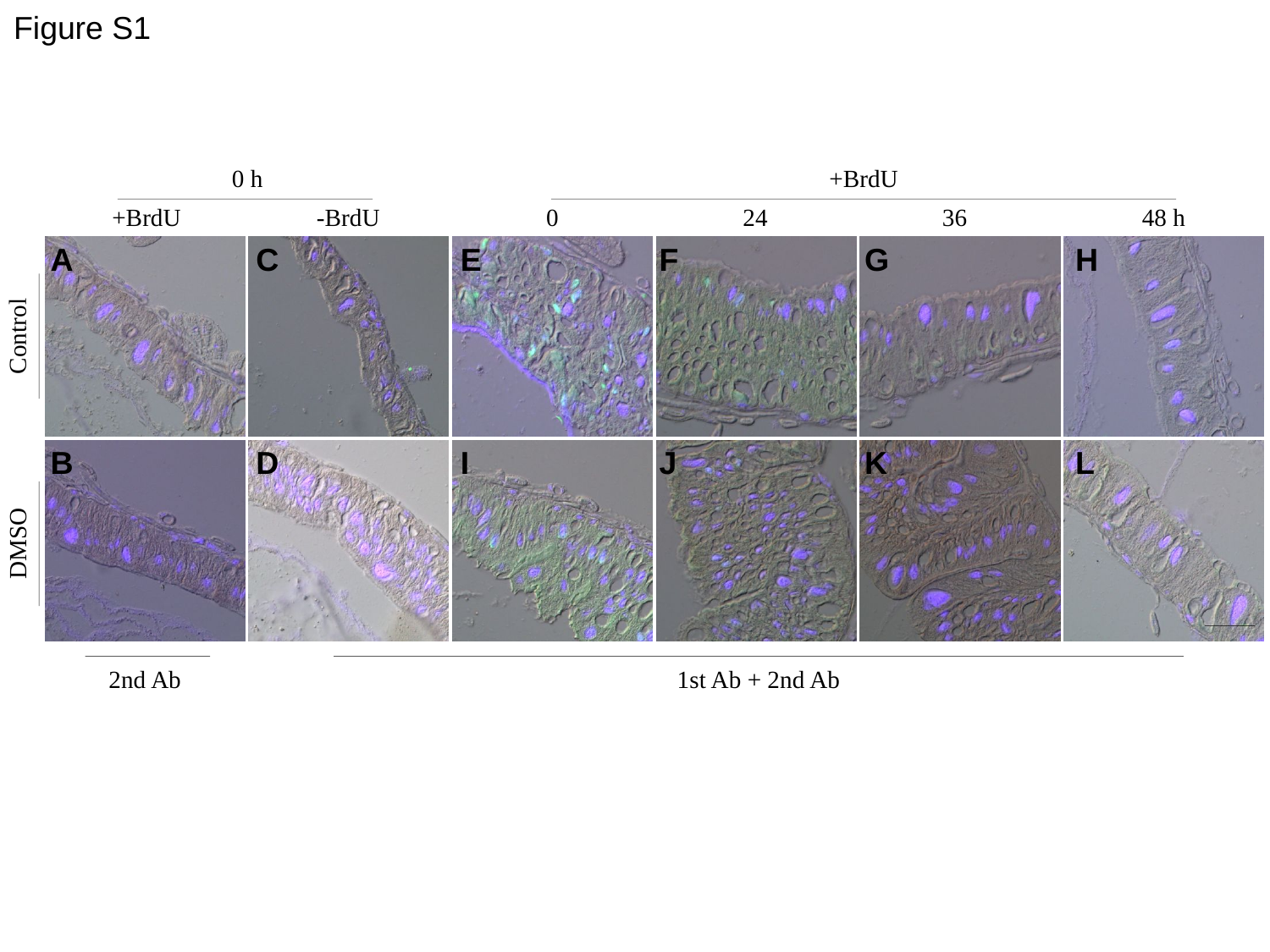

Figure S1
+BrdU
0 h
+BrdU
-BrdU
0
24
36
48 h
Control
DMSO
2nd Ab
1st Ab + 2nd Ab
A
C
E
F
G
H
B
D
I
J
K
L

Supplement: Additional file 3: Figure S1. — DMSO did not increase BrdU incorporation according to observation by microscopy. DMSO was the solvent for 20E. As a control, the same volume of DMSO (20 %) was injected and the midguts were sampled. As another control, naïve and DMSO-injected larvae were also sampled at 0 h post injection. Some samples of naïve larvae (without 20E injection) and DMSO-injected larvae were stained with secondary (2nd) antibodies only (A-B). DMSO did not enhance BrdU incorporation (E-L). Without BrdU injection or by omitting the primary (1st) antibody against BrdU, there was no signal (A-D). Bar: 50 μm. (PPT 3272 kb) [file 12864_2016_3162_MOESM3_ESM.ppt]

## Slide 1
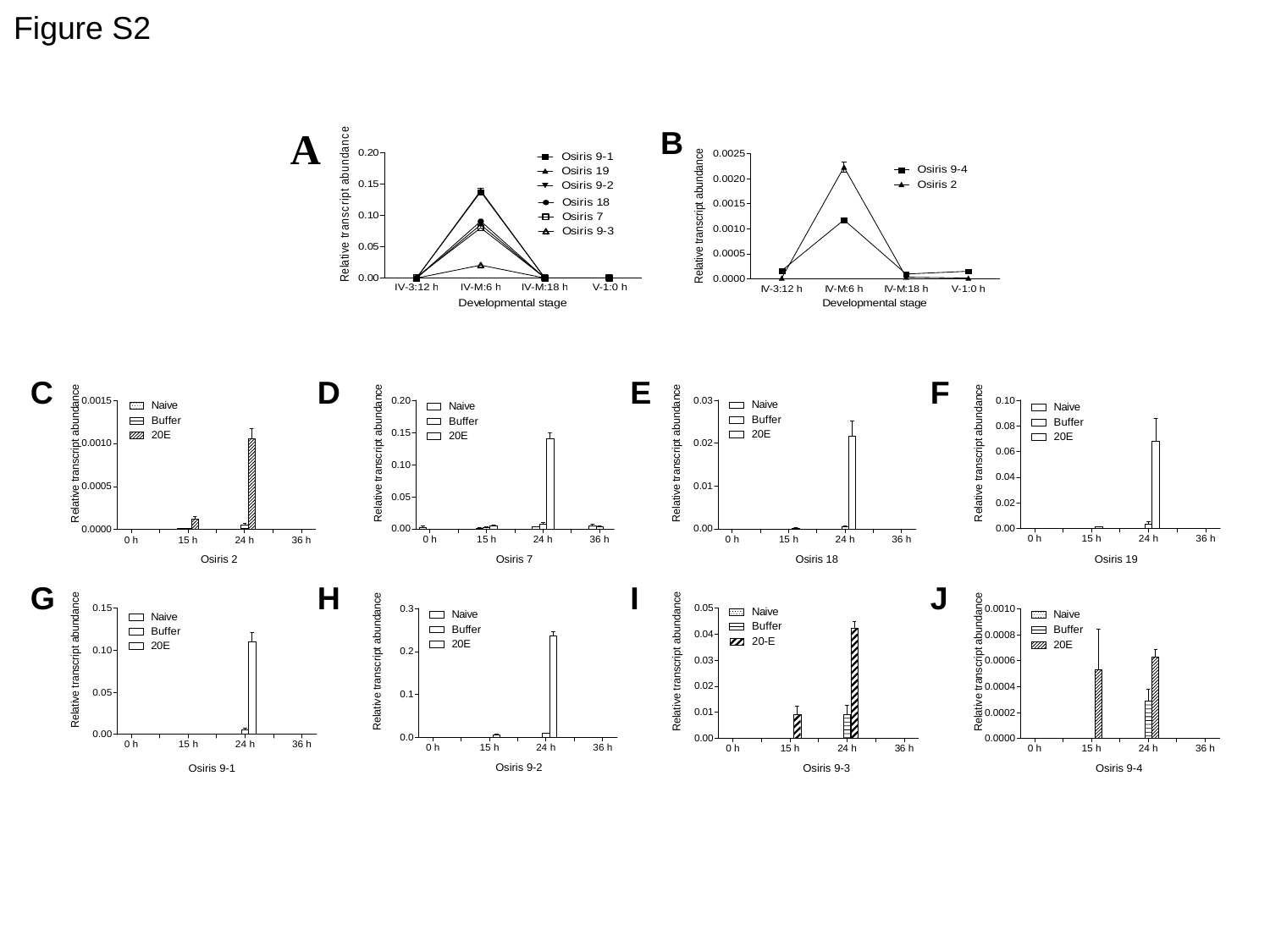

Figure S2
A
B
C
D
E
F
G
H
I
J

Supplement: Additional file 5: Figure S2. — 20E increased the expression of osiris genes in the midgut. 20E injection was performed as described in methods. (A–B) Expression of osiris genes during the indicated developmental stages as assayed by qRT-PCR. Osiris genes assayed here were up-regulated at the early molting stage (6 h after the initiation of molting). (C–J) 20E injection induced the expression of osiris genes in the midgut. All osiris genes were up-regulated at 24 h post 20E injection. The changes were not obvious at other time points except Osiris 9-4 at 15 h post 20E injection. (PPT 330 kb) [file 12864_2016_3162_MOESM5_ESM.ppt]
