# Supplementary material for: Analysis of gene expression in the midgut of Bombyx mori during the larval molting stage
Source: BMC Genomics. 2016 Nov 3;17:866. doi: 10.1186/s12864-016-3162-8 (PMC5096333; doi:10.1186/s12864-016-3162-8)
Supplement: Additional file 4: Table S3. — List of cuticle proteins in the midguts of the molting stage after identification by LC-MS/MS. The gel bands were excised as shown in Fig. 7A-a. (PPT 106 kb) [file 12864_2016_3162_MOESM4_ESM.ppt]

## Slide 1
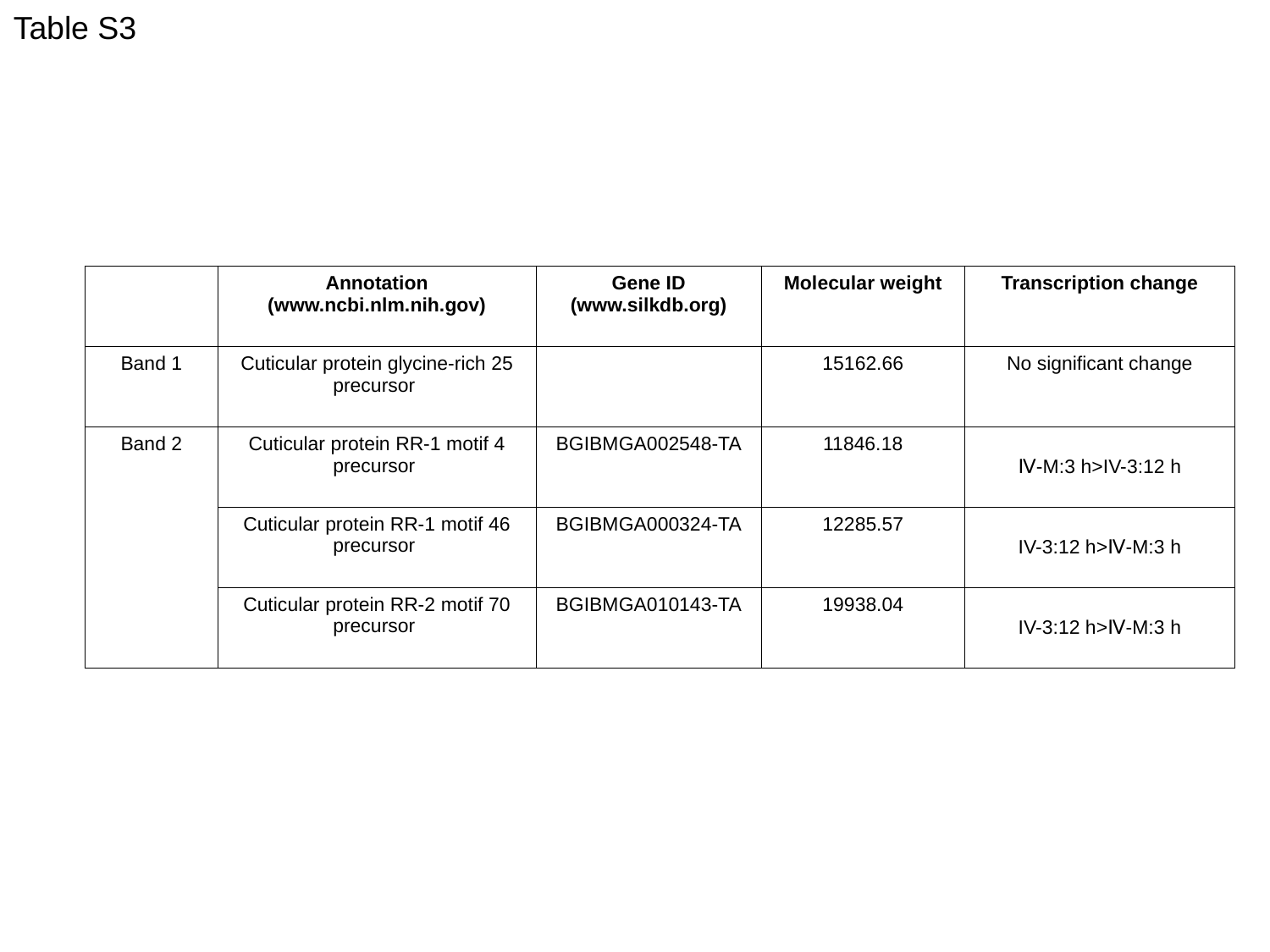

Table S3
| | Annotation (www.ncbi.nlm.nih.gov) | Gene ID (www.silkdb.org) | Molecular weight | Transcription change |
| --- | --- | --- | --- | --- |
| Band 1 | Cuticular protein glycine-rich 25 precursor | | 15162.66 | No significant change |
| Band 2 | Cuticular protein RR-1 motif 4 precursor | BGIBMGA002548-TA | 11846.18 | Ⅳ-M:3 h>IV-3:12 h |
| | Cuticular protein RR-1 motif 46 precursor | BGIBMGA000324-TA | 12285.57 | IV-3:12 h>Ⅳ-M:3 h |
| | Cuticular protein RR-2 motif 70 precursor | BGIBMGA010143-TA | 19938.04 | IV-3:12 h>Ⅳ-M:3 h |
